# Why Skin Carotenoid Measurements Cannot Serve as a Proxy for Macular Pigment Optical Density (MPOD): A Biochemical, Anatomical, Optical, and Statistical Review

**DOI:** 10.3390/nu18030492

**Published:** 2026-02-02

**Authors:** Mohsen Sharifzadeh

**Affiliations:** Electrical & Computer Engineering Department, Brigham Young University (BYU), Provo, UT 84602, USA; sharifzadeh.mohsen@gmail.com or msh1344@byu.edu

**Keywords:** macular pigment optical density (MPOD), skin carotenoids, lutein, zeaxanthin, meso-zeaxanthin, melanin, retinal imaging, biomarker specificity, nutrition and eye health

## Abstract

Carotenoids accumulate in both the skin and the macula, but their biochemical specificity, anatomical localization, optical environments, and temporal kinetics differ fundamentally. Despite superficial similarities, these distinctions raise questions about whether non-invasive skin carotenoid measurements, which are obtained using reflection spectroscopy or resonance Raman spectroscopy, can meaningfully reflect macular pigment optical density (MPOD), a retina-specific biomarker associated with visual performance and neuroprotective function. This review synthesizes evidence across biochemistry, tissue distribution, optical pathways, kinetic behavior, and statistical correlations to evaluate this proposed relationship. Skin carotenoid measurements capture a broad mixture of dietary carotenoids, which are dominated by β-carotene and lycopene, that accumulate superficially within the epidermis and dermis and respond rapidly to short-term dietary and environmental changes. In contrast, MPOD reflects only lutein, zeaxanthin, and meso-zeaxanthin, which are selectively transported into the foveal neurosensory retina and change slowly through regulated retinal uptake and deposition. Across human studies, correlations between skin carotenoids and MPOD are weak, inconsistent, and biologically implausible, with large cohort analyses demonstrating near-zero associations. Collectively, evidence across biochemical, anatomical, optical, physiological, and statistical domains shows that skin carotenoid values encode general systemic antioxidant exposure, whereas MPOD reflects a highly localized, retina-specific carotenoid reservoir. Therefore, skin carotenoid measurements cannot be used to estimate, substitute for, or infer macular pigment levels. Accurate assessment of MPOD requires direct retinal imaging technologies.

## 1. Introduction

Carotenoids are lipid-soluble pigments with essential biological functions, including antioxidant protection, modulation of oxidative stress, and support of cellular and mitochondrial activity. Two tissues where carotenoids accumulate in measurable quantities are the skin and the macula. Although both reflect aspects of systemic carotenoid exposure, their biochemical composition, optical environments, physiological roles, and response kinetics differ fundamentally.

Skin carotenoids, measured non-invasively using reflection spectroscopy or resonance Raman spectroscopy, primarily represent β-carotene, lycopene, α-carotene, and β-cryptoxanthin. Their concentrations are influenced by dietary patterns, lifestyle factors, UV exposure, and systemic oxidative stress. As summarized in Table 1, the skin exhibits a broad and non-selective carotenoid profile.

The macula, by contrast, is a specialized retinal region that selectively accumulates lutein, zeaxanthin, and meso-zeaxanthin. These carotenoids play critical roles in blue-light filtration, quenching of reactive oxygen species, and protecting both photoreceptors and the retinal pigment epithelium (RPE). Their distribution within specific inner retinal layers, spatially distinct from RPE melanin, has been well described in the literature [1]. MPOD is therefore widely used in clinical and research settings to assess retinal health, age-related macular degeneration (AMD) risk, and visual performance.

Because both skin and macular carotenoids are influenced by diet, some studies and certain commercial devices have proposed using skin carotenoid scores as surrogate markers for MPOD. Although intuitively appealing, this assumption lacks biological, anatomical, optical, and statistical justification.

Skin carotenoid levels and MPOD reflect fundamentally different physiological systems governed by distinct transport proteins, carotenoid selectivity, spatial distribution patterns, and temporal response behaviors. Correlations between the two biomarkers are weak to moderate at best, and statistical association alone does not establish biological equivalence or justify surrogate substitution.

### Objective

This review provides a rigorous, domain-by-domain evaluation of whether skin carotenoid measurements can serve as proxies for macular pigment optical density. By synthesizing biochemical, anatomical, optical, physiological, and statistical evidence, we demonstrate why direct retinal imaging remains the only accurate method for assessing MPOD.

## 2. Biochemical and Physiological Distinctions Between Skin and Macular Carotenoids

Carotenoid distribution in the human body is tissue-specific and shaped by local physiological demands and transport-protein expression. This selectivity is a foundational reason why skin carotenoid measurements cannot be used to infer macular pigment content.

### 2.1. Skin vs. Macular Carotenoids

#### 2.1.1. Skin Carotenoids

The skin contains a broad mixture of dietary carotenoids, dominated by β-carotene, lycopene, α-carotene, and β-cryptoxanthin, with only minor contributions from lutein and zeaxanthin [2,3]. These compounds reflect general fruit-and-vegetable intake and circulate widely throughout the body. Importantly, the carotenoids most abundant in skin, such as β-carotene and lycopene, do not contribute to macular pigment formation.

#### 2.1.2. Macular Carotenoids

The macula selectively accumulates lutein, zeaxanthin, and meso-zeaxanthin, three carotenoids essential for retinal protection and visual function. Their uptake is mediated by specialized transport proteins such as SCARB1, STARD3, and GSTP1, which preferentially deliver these molecules to the foveal neurosensory retina [4,5]. No other dietary carotenoids can substitute for these pigments. Table 1 summarizes the qualitative tissue specificity of major carotenoids and highlights a central mismatch: skin measurements reflect a broad, non-selective carotenoid mixture dominated by β-carotene and lycopene, whereas the macula reflects selective xanthophyll accumulation. This biochemical divergence alone precludes the use of skin carotenoid scores as surrogates for MPOD.

### 2.2. Why Carotenoid Selectivity Matters

Because the skin and retina retain entirely different carotenoid species, changes in skin carotenoid levels cannot be interpreted as changes in macular pigment. Increases in skin carotenoids are largely driven by β-carotene and lycopene, which are irrelevant to MPOD, while the retina responds exclusively to lutein, zeaxanthin, and meso-zeaxanthin.

Dietary intervention studies make this distinction evident. Tomato juice, carrot-based foods, and mixed-carotenoid formulations substantially elevate skin carotenoid scores due to their high β-carotene and lycopene content, yet produce little or no change in MPOD because the retina requires xanthophyll-specific intake. Conversely, meaningful increases in MPOD typically occur only after sustained consumption of lutein and zeaxanthin.

Thus, skin carotenoid measurements reflect broad systemic antioxidant exposure, whereas MPOD reflects selective carotenoid deposition in neural tissue. Biochemical selectivity alone makes skin carotenoid values unsuitable as proxies for MPOD.

### 2.3. Differing Physiological Roles

Carotenoids serve distinct biological functions in the skin and retina.

#### 2.3.1. Skin

In the skin, carotenoids act as general antioxidants, neutralizing reactive oxygen species generated by UV exposure, environmental pollutants, and metabolic stress. Because this role is diffuse and non-specific, skin carotenoid levels fluctuate readily with environmental and systemic conditions.

#### 2.3.2. Retina

In the macula, carotenoids serve specialized neural functions, including blue-light absorption, quenching of photochemically generated radicals, protection of photoreceptors and the RPE, and enhancement of visual performance. These functions require selective retention, high local concentration, and integration within retinal architecture, all of which are absent in skin tissue.

### 2.4. Temporal Response Differences

The temporal dynamics of carotenoid accumulation differ markedly between the skin and retina. Skin carotenoids rise rapidly, typically within 2–4 weeks, because the epidermis and superficial dermis turn over quickly and respond immediately to dietary β-carotene and lycopene. Levels typically plateau within 4 to 8 weeks, although the exact timing varies among individuals and is influenced by dietary intake.

By contrast, MPOD changes slowly. Measurable increases typically appear only after 8–12 weeks of targeted supplementation with lutein and zeaxanthin and may require 3–6 months or longer. This delayed response reflects selective transport, deeper anatomical deposition, and the slow turnover of retinal tissue. Across studies measuring both tissues, skin carotenoids display early, rapid increases, whereas MPOD exhibits delayed, gradual rises that continue for months [3,6,7].

### 2.5. Summary of Biochemical and Physiological Arguments

Skin and macular tissues process carotenoids through fundamentally different mechanisms. The skin accumulates a broad mix of dietary carotenoids dominated by β-carotene and lycopene, while the macula selectively retains lutein, zeaxanthin, and meso-zeaxanthin via specialized transport proteins. Skin carotenoid levels respond rapidly to dietary intake and environmental influences, whereas MPOD changes only with sustained xanthophyll intake and exhibits slow, regulated accumulation.

Therefore, skin carotenoid measurements reflect general nutritional and antioxidant status but provide no insight into retinal carotenoid deposition or macular pigment levels. These biochemical and physiological distinctions form a central rationale for why skin carotenoid scores cannot serve as proxies for MPOD.

## 3. Anatomical and Optical Differences Between Skin and Macular Pigment

The anatomical location, structural organization, and optical environments of carotenoids in the skin and retina differ profoundly. These differences alone are sufficient to invalidate the idea that a skin carotenoid score could predict MPOD. Whereas MPOD is highly localized, spatially heterogeneous, and shaped by retinal-specific optical properties, skin carotenoids are broadly distributed, optically mixed, and influenced by entirely different tissue-layer structures.

### 3.1. Sampling Area and Spatial Organization: 3 mm vs. 2 mm, Uniform vs. Localized

Non-invasive skin carotenoid devices typically measure a relatively large optical sampling volume of approximately 3–4 mm, capturing signals from the stratum corneum, epidermis, and upper dermis [1,8]. Within these superficial layers, carotenoid distribution is diffuse and relatively uniform, reflecting systemic dietary exposure rather than any tissue-specific specialization. Because of this broad sampling region, skin carotenoid measurements inherently average carotenoid content over a wide area, rather than isolating highly localized accumulation.

By contrast, macular pigment is concentrated almost entirely within the central 1–2 mm of the fovea, the region responsible for the highest visual acuity, fine color discrimination, and peak photoreceptor density. The macular pigment distribution exhibits a sharply peaked profile that is anatomically and functionally distinct from the deeper and more diffuse distribution of RPE melanin, as described in prior anatomical imaging studies [8]. Macular pigment distribution also varies significantly among individuals, often presenting as a central peak, ring, low-central-density, or asymmetric patterns, all of which have been documented in the literature [3]. These spatial phenotypes carry both functional and clinical significance.

Because skin devices sample a broad, uniform surface region, a single skin carotenoid score cannot capture the localized foveal concentration of macular pigment, the relative balance between central and parafoveal pigment, or the individual spatial patterns commonly observed in the retina. MPOD is inherently a spatially localized retinal biomarker; skin carotenoid scores, by design, cannot resolve or approximate these spatial properties.

### 3.2. Layered Optical Environments: Epidermis vs. Multilayered Retina

The optical pathways governing carotenoid measurement in the skin and retina differ fundamentally.

In reflection spectroscopy and resonance Raman spectroscopy, the signal detected from the skin arises primarily from superficial tissue layers. Photons interact with epidermal melanin, hemoglobin in the superficial dermal vasculature, collagen-associated scattering structures, and carotenoids located near the surface. Because these interactions occur within the first few hundred micrometers of tissue, skin measurements are inherently surface-weighted. The resulting optical signal represents a mixture of chromophores rather than a distinct carotenoid layer, limiting the ability to isolate carotenoid-specific absorption or scattering.

By contrast, MPOD can only be assessed by imaging light that passes through multiple ocular and retinal layers, each contributing its own optical signature [1,8]. Incident light travels through the cornea, crystalline lens, and vitreous before reaching the neurosensory retina. The return signal is shaped by interactions with macular pigment in the inner retina, the densely melanized RPE, choroidal vasculature, and RPE lipofuscin. These layered structures introduce wavelength-dependent absorption, substantial melanin attenuation, and complex scattering behavior. Age-related lens changes further alter short-wavelength transmission. As documented in prior anatomical imaging studies, macular pigment lies anterior to the RPE melanin layer, creating a multilayered environment that requires specialized correction algorithms for accurate MPOD isolation [8].

Because skin spectroscopy captures only surface-biased signals dominated by melanin and hemoglobin, while MPOD imaging requires navigating a deeply layered optical system, no superficial measurement can reconstruct the retinal optical pathway. Direct retinal imaging is therefore indispensable for accurate MPOD quantification.

### 3.3. Melanin Distribution: Uniform in Skin vs. Concentrated in the Fovea

Melanin distribution represents another major optical distinction between skin and retina. In the skin, melanin is broadly and relatively uniformly distributed within the epidermis. Although concentrations vary across Fitzpatrick skin types, the distribution remains diffuse rather than spatially localized. As a result, melanin provides a relatively stable background absorption in skin spectroscopy.

In contrast, retinal melanin is densely concentrated beneath the fovea in the RPE. This melanin layer is spatially co-localized with the macular region used for MPOD assessment [8] and strongly absorbs both excitation and emission wavelengths. Its influence is substantial, varies across individuals, and interacts directly with macular pigment absorption.

Accurate MPOD measurement requires consideration of foveal melanin absorption, a factor that is unique to the retinal environment and has no analogue in skin-based measurements, where melanin is anatomically separate from the structures relevant to macular pigment.

### 3.4. Hemoglobin and Vascular Influences

Hemoglobin affects optical measurements in both skin and retina, but its anatomical location, temporal variability, and interaction with carotenoid signals differ fundamentally between the two tissues. These differences, which have direct implications for measurement validity, are addressed in detail in Section 5.2.

### 3.5. Fundamental Optical Non-Equivalence

Taken together, the optical environments of the skin and retina are fundamentally non-equivalent. Skin-based devices capture surface-weighted signals dominated by epidermal melanin, dermal hemoglobin, and superficial carotenoid scattering. MPOD imaging, however, isolates a deeply embedded pigment and must disentangle macular pigment absorption from RPE melanin, lipofuscin autofluorescence, and the multilayered neural architecture of the retina.

In the skin, melanin is diffuse and uniform; in the retina, it is densely concentrated beneath the fovea. Skin carotenoid signals are optically mixed with hemoglobin and shallow scattering, while MPOD signals are shaped by deep melanin absorption, lipofuscin fluorescence, retinal curvature, and layer-specific photon paths.

These distinctions reflect fundamental optical non-equivalence. Skin carotenoid measurements capture general antioxidant presence in superficial tissue, whereas MPOD quantifies a specialized protective pigment embedded within the macula. Because these systems operate under entirely different optical constraints, geometries, and chromophore interactions, no skin-based measurement can replicate or approximate the optical signatures required to determine MPOD.

### 3.6. Conclusion of Anatomical and Optical Section

The anatomical localization and optical environment of macular pigment differ so substantially from those of skin carotenoids that the two measurements cannot be equated or interpreted interchangeably. Skin measurements lack the spatial resolution, optical corrections, tissue specificity, and physiological relevance required for MPOD assessment. Because MPOD arises from a highly localized neural pigment embedded within multilayer retinal structures, and because skin carotenoids are distributed diffusely within superficial epidermal and dermal layers, skin carotenoid scores cannot serve as proxies for macular pigment optical density.

## 4. Temporal Kinetics: Skin Carotenoids Respond Quickly While MPOD Changes Slowly

The temporal behavior of carotenoids in different tissues provides strong biological evidence that skin carotenoid scores and MPOD reflect fundamentally distinct processes. Even under identical dietary interventions, the two tissues exhibit markedly different response trajectories due to differences in tissue structure, turnover rate, metabolic function, and carotenoid transport mechanisms. These kinetic disparities make it biologically implausible for skin carotenoid measurements to serve as surrogate indicators of MPOD.

### 4.1. Skin Carotenoids Increase Rapidly (Weeks)

Intervention studies consistently demonstrate that skin carotenoids rise quickly, often within 2 to 4 weeks, in response to increased consumption of carotenoid-rich foods [3,7]. This rapid change is driven primarily by β-carotene and lycopene, which dominate the skin’s carotenoid profile and accumulate readily in the epidermis and superficial dermis. Fast epidermal turnover and continual exposure to oxidative stress, sunlight, and environmental factors further contribute to the dynamic behavior of skin carotenoid levels.

Both reflection spectroscopy and resonance Raman spectroscopy studies show a characteristic kinetic pattern that includes a significant increase by about week 4, followed by a plateau between weeks 8 and 12, even with continued supplementation [7]. These methods also respond strongly to carotenoids such as lycopene, which do not contribute to macular pigment. Overall, skin carotenoid levels display a rapid early rise and then stabilize, functioning as short-term indicators of dietary intake and systemic antioxidant exposure rather than tissue-specific markers of retinal carotenoid status.

### 4.2. MPOD Increases Slowly (Months to Years)

In contrast, macular pigment optical density increases only after sustained, targeted ingestion of lutein, zeaxanthin and meso-zeaxanthin, and requires substantially longer to show measurable change. Human supplementation studies consistently demonstrate that MPOD generally shows minimal or no detectable increase within the first 8 weeks, followed by gradual rises over approximately 4–6 months, with significant improvements often requiring 6–12 months of continuous intake [6,7].

This slow kinetic profile reflects retinal biology: macular carotenoids are selectively taken up via transport proteins such as SCARB1, STARD3, and GSTP1 [4], deposited within the neurosensory retina, and maintained with low turnover because of their essential roles in blue-light filtration and oxidative protection. Unlike the epidermis, where carotenoids are replenished rapidly, retinal carotenoid incorporation depends on neural tissue metabolism and regulated deposition pathways. Longitudinal MPOD studies typically show minimal early change followed by gradual, sustained increases over extended periods [6]. MPOD therefore represents a long-term biomarker of cumulative retinal carotenoid deposition rather than a short-term indicator of dietary fluctuation.

### 4.3. Comparative Supplementation Studies: Divergence of Skin and MPOD Timelines

Studies measuring both skin carotenoids and MPOD in the same subjects provide some of the strongest evidence of temporal non-equivalence between the two biomarkers. Across multiple controlled supplementation trials, the time-dependent responses of skin and retinal carotenoids diverge in consistent and biologically meaningful ways [3,7].

Skin carotenoids show significant increases by week 4, even when the intervention includes mixed carotenoids that do not influence the macula, whereas MPOD shows no measurable change at week 4 regardless of dose. Skin carotenoids typically plateau by week 8 because of rapid epidermal turnover and shallow sampling depth, while MPOD generally shows minimal or no detectable change within the first 8 weeks and begins to rise only with longer-term supplementation.

These divergent trajectories have been documented across studies that measured both skin carotenoids and MPOD in the same individuals [6,7]. Even under identical supplementation protocols, the two tissues follow fundamentally different temporal patterns.

Together, these findings reinforce three central points:

(1) Different carotenoid classes dominate each tissue (lycopene and β-carotene in skin vs. lutein, zeaxanthin, and meso-zeaxanthin in the macula);
(2) Turnover rates differ profoundly, with skin responding within weeks and the macula responding over months;
(3) Tissue depth and metabolic handling diverge, with skin reflecting superficial, rapidly changing antioxidant pools, and the retina reflecting slow, regulated accumulation within the neurosensory retinal layers.

Thus, even under tightly controlled dietary interventions, the time courses of skin carotenoids and MPOD do not overlap, demonstrating that skin carotenoid measurements cannot predict retinal carotenoid accumulation.

### 4.4. Why Temporal Mismatch Proves Non-Equivalence

The opposing temporal dynamics of skin carotenoids and MPOD highlight fundamental biological differences between the two tissues. Skin carotenoid levels rise rapidly because the epidermis responds quickly to circulating β-carotene and lycopene and exhibits fast cellular turnover. MPOD, however, increases slowly and only in response to sustained intake of lutein, zeaxanthin and meso-zeaxanthin. These carotenoids must be selectively transported, deposited within deeper retinal layers, and incorporated into neural tissue, processes that unfold over months rather than weeks.

This temporal mismatch is further emphasized by the fact that skin carotenoids increase robustly in response to carotenoids that have no influence on macular pigment. Foods rich in β-carotene or lycopene can markedly elevate skin carotenoid scores yet produce no measurable change in MPOD. Meanwhile, MPOD is governed by retina-specific transport mechanisms (SCARB1, STARD3, GSTP1) and depends on selective xanthophyll uptake rather than broad carotenoid exposure.

Consequently, even if a correlation appears at a single time point, skin carotenoid scores cannot track, predict, or substitute for MPOD changes over time. The two biomarkers operate on different timelines, respond to different carotenoid classes, and reflect distinct physiological functions, making temporal substitution biologically implausible.

### 4.5. Conclusion of Temporal Kinetics

- Skin carotenoids respond rapidly to dietary intake, typically increasing within 2–4 weeks and plateauing within 6–8 weeks.
- MPOD changes slowly and requires sustained intake of lutein, zeaxanthin, and meso-zeaxanthin over months.
- Skin carotenoid dynamics are driven by superficial tissue turnover and broad carotenoid exposure, whereas MPOD reflects regulated retinal uptake and slow neural deposition.
- Identical supplementation protocols produce divergent time courses in skin and retina.
- Because their temporal trajectories do not align, skin carotenoid measurements cannot track, predict, or substitute for MPOD.

## 5. Why Direct Retinal Imaging Is Necessary for MPOD: Structural and Optical Differences Between Skin and Retina

Accurate assessment of MPOD cannot be derived from any external tissue because the anatomical and optical environments of the retina differ fundamentally from those of the skin. The macula is a multilayered neural structure with highly localized pigment deposition, deep melanin absorption, and complex light-tissue interactions that require dedicated imaging methods. In contrast, skin carotenoid measurements capture superficial, diffuse signals influenced by epidermal melanin, dermal hemoglobin, and environmental factors. These structural and optical mismatches make it impossible for a skin carotenoid score to approximate or infer MPOD. This section outlines the key distinctions that demonstrate why MPOD is a strictly retinal biomarker requiring direct retinal imaging.

### 5.1. Melanin Distribution: Uniform in Skin vs. Highly Concentrated in the Fovea

Melanin plays fundamentally different anatomical and optical roles in the skin and retina, creating measurement constraints that cannot be bridged by any skin-based technique.

#### 5.1.1. Skin Melanin

In the skin, melanin is

- Broadly distributed across the epidermis;
- Relatively uniform within a given region (such as the palm or fingertip);
- Spatially separate from carotenoid-containing layers;
- A stable background absorber for reflection and Raman spectroscopy.

Because epidermal melanin does not overlap with carotenoid deposition, its influence on optical measurements is relatively consistent and can be corrected with standard models.

#### 5.1.2. Retinal Melanin

In the retina, melanin is anatomically and optically more complex. It is

- Extremely concentrated beneath the fovea within the RPE [8];
- Directly co-located with the region used for MPOD measurement;
- Highly absorbent at the excitation and emission wavelengths used for MPOD imaging;
- Variable across individuals and in disease states;
- A major determinant of apparent MPOD magnitude, including in imaging systems that attempt to model its influence.

#### 5.1.3. Implication

Because retinal melanin spatially overlaps with macular pigment and strongly influences the detected retinal signal, any method that does not image the retina directly cannot account for the interaction between melanin and macular pigment. Skin optical measurements never encounter RPE melanin and therefore cannot incorporate this retinal-specific feature into their analysis. For this reason, accurate MPOD quantification requires direct retinal imaging.

### 5.2. Hemoglobin: Dermal Vasculature vs. Choroidal Vasculature

Hemoglobin exerts fundamentally different optical influences in skin compared with the retina.

#### 5.2.1. Skin

In the skin, hemoglobin is contained in the superficial and mid-dermal vasculature. Its absorption properties strongly affect reflection-based measurements, and the signal varies with blood flow, temperature, external pressure, autonomic activity, and emotional state. Because hemoglobin absorption overlaps with the wavelengths used for carotenoid assessment, fluctuations in dermal perfusion introduce variability unrelated to true carotenoid concentration.

#### 5.2.2. Retina

In the retina, hemoglobin is located primarily within the choroidal vasculature, beneath both the neurosensory retina and the melanin-rich RPE. Consequently, its optical influence is filtered through RPE melanin and interacts with lipofuscin autofluorescence rather than contributing directly to the macular pigment signal. Retinal imaging systems use wavelength-dependent modeling to help distinguish hemoglobin effects from macular pigment absorption, an approach that is specific to retinal anatomy and not applicable to skin spectroscopy.

#### 5.2.3. Implication

Because hemoglobin interacts with light through entirely different anatomical layers and optical pathways in skin and retina, its influence cannot be treated equivalently across these tissues. This mismatch alone precludes the use of skin-based optical measurements for quantifying macular pigment.

### 5.3. Sampling Depth: Superficial Skin Layers vs. Multilayered Retina

Skin-based carotenoid measurements probe only the most superficial tissue layers. Reflection spectroscopy and Raman techniques typically interrogate the stratum corneum, epidermis, and upper dermis, with sampling depths on the order of 200–300 μm. Within this shallow region, carotenoids are intermixed with dominant chromophores (epidermal melanin, dermal hemoglobin) and scattering structures associated with collagen. Variability in skin hydration, epidermal thickness, and barrier properties further modifies light penetration and the effective sampling volume. As a result, the detected signal represents a mixed optical contribution from heterogeneous superficial layers rather than a discrete carotenoid compartment.

By contrast, MPOD imaging interrogates a deeply layered optical system. Incident light must traverse multiple neurosensory retinal layers before interacting with macular pigment, and the returning fluorescence or reflectance must pass through RPE, lipofuscin, and the choroid. In clinical settings, ocular media such as the crystalline lens can introduce additional absorption, particularly in the presence of cataract, requiring wavelength selection and correction strategies tailored to the retinal environment.

Because each retinal layer exhibits distinct absorption and scattering properties, MPOD quantification requires depth-dependent, wavelength-specific modeling to isolate macular pigment from coexisting chromophores. None of these anatomical or optical interactions occurs in the skin, and no superficial measurement can reconstruct the retinal light path.

#### Implication

The retina’s multilayered optical architecture is fundamentally incompatible with the shallow sampling and mixed-signal characteristics of skin spectroscopy. Skin carotenoid measurements cannot approximate or infer MPOD, which is embedded within deep neural and pigment epithelial layers inaccessible to external optical methods.

### 5.4. Tissue Geometry and Optical Pathways

#### 5.4.1. Skin

The skin is a predominantly scattering, surface-weighted optical tissue. In this environment:

- Most detected photons originate from very shallow depths;
- Carotenoids reside above major chromophores such as hemoglobin;
- Optical paths are short and heavily influenced by epidermal and superficial dermal properties.

Skin-based optical measurements, therefore, primarily capture superficial carotenoid signals mixed with melanin and vascular influences.

#### 5.4.2. Retina

The retina operates as a complex transmission-reflection hybrid system:

- Incident light passes through multiple neural layers before returning to the detector;
- Deep absorption occurs due to RPE melanin and lipofuscin;
- Macular pigment is embedded within tightly organized neural layers;
- The geometry of the retina is curved and layered;
- The spatial distribution of macular pigment varies substantially across individuals [1].

These characteristics produce intricate depth-dependent interactions that cannot be reproduced by any superficial optical measurement.

#### 5.4.3. Implication

Because the geometry, depth, and optical pathways of the retina are fundamentally different from those of the skin, skin carotenoid measurements cannot recreate or approximate the optical interactions that define MPOD imaging. Only direct retinal imaging can capture these neural and pigment-specific processes.

### 5.5. Why These Differences Require Direct Retinal Imaging

Taken together, the distinctions in melanin distribution, hemoglobin localization, sampling depth, and optical geometry demonstrate that the skin and retina operate in fundamentally different optical domains.

- The skin is a diffuse, uniform, superficial medium in which the measured carotenoid signal comes primarily from β-carotene and lycopene in the stratum corneum and superficial dermis.
- The retina is a multilayered neural structure containing densely packed macular pigment, high concentrations of RPE melanin, and deep choroidal vasculature, each of which shapes the optical pathways used to measure MPOD.

Because these retinal features determine both the magnitude and spectral characteristics of MPOD signals, no superficial optical measurement of the skin can reproduce or approximate them. Therefore, accurate quantification of macular pigment optical density requires direct retinal imaging. Skin carotenoid scores cannot substitute for, estimate, or infer MPOD under any optical, anatomical, or physiological framework.

## 6. Statistical Evidence: Weak, Inconsistent, and Biologically Implausible Correlations Between Skin Carotenoids and MPOD

Although skin and retinal tissues differ biochemically, anatomically, and physiologically, one could still ask whether statistical correlations might justify using skin carotenoid measurements as predictors of MPOD. For this reason, multiple studies across diverse populations have examined the relationship between these biomarkers. The collective evidence is unequivocal: skin carotenoid scores demonstrate weak, inconsistent, and biologically implausible correlations with MPOD, and therefore cannot serve as surrogate retinal biomarkers. This finding is consistent across pediatric and adult cohorts, small and large population samples, and across multiple measurement modalities.

This section summarizes the statistical evidence and explains why the observed correlations fail under biological and methodological scrutiny.

### 6.1. Wide Range of Reported Correlations (r = 0.02 to 0.66)

Published studies report correlations between skin carotenoid levels and MPOD that range from negligible to moderate (r = 0.02–0.66) [9,10,11,12,13]. Large population-based studies consistently show near-zero associations. For example, pediatric cohorts exceeding 350 subjects yield essentially no relationship between the two biomarkers (r ≈ 0.02), and adult cohorts close to 1000 subjects show only very weak correlations (r ≈ 0.07).

A small number of narrowly defined adult studies have reported moderate correlations (r = 0.4–0.66), but these typically involve homogeneous populations with limited dietary and ethnic variability and rely on single measurement techniques, limiting generalizability.

From a biomarker science perspective, correlations below r = 0.8 are insufficient to justify surrogate or interchangeable use, particularly when the underlying biological pathways differ fundamentally. Even the strongest reported values fall far below the threshold required for substituting skin carotenoid measurements for MPOD.

### 6.2. Large Studies Show the Weakest Correlations

Across the literature, the largest and most methodologically rigorous studies consistently demonstrate the weakest associations between skin carotenoid scores and MPOD, a hallmark of biological non-equivalence.

Pediatric Cohort (*n* > 350)

- r ≈ 0.02.
- *p* = nonsignificant.
- No measurable relationship between the two biomarkers.

Large Japanese Adult Cohort (*n* ≈ 985)

- r ≈ 0.07.
- statistical significance attributable to a large sample size.
- r^2^ ≈ 0.005, explaining only 0.5% of MPOD variance.

These findings contrast with moderate correlations sometimes reported in small studies, which can be artificially inflated by limited dietary diversity, narrow age or ethnicity ranges, reduced biological variability, sampling bias, or reliance on a single measurement modality. When evaluated in large, diverse populations, correlations collapse toward zero, indicating that any apparent signal in smaller datasets does not generalize and lacks biological validity.

### 6.3. Methodological Variation Produces Artificial Correlations

Differences in methodology contribute substantially to the variability of reported correlations. Variation arises from differences in

- MPOD measurement modality (heterochromatic flicker photometry, autofluorescence imaging, reflectometry).
- wavelength specificity and optical design.
- subject age, pigmentation, and skin physiology.
- dietary habits and carotenoid profiles.
- melanin and hemoglobin interference.
- transport mechanisms influencing lutein/zeaxanthin uptake.
- supplement composition and dosing schedules.

Critically, skin devices quantify total carotenoids, dominated by β-carotene and lycopene, whereas MPOD quantifies only lutein, zeaxanthin, and meso-zeaxanthin. Correlating two unrelated sets of carotenoid molecules in two biologically distinct tissues produces unstable associations, which the literature repeatedly reflects.

### 6.4. Correlation Does Not Imply Interchangeability

Even the most optimistic small-sample correlations (r = 0.5–0.66) cannot justify using skin carotenoid values as surrogates for MPOD.

A. Correlated biomarkers cannot substitute for one another

Correlation measures co-movement, not equivalence.

For two biomarkers to be interchangeable, validation studies typically require:

- Strong correlations (often in the range of r = 0.7–0.9);
- Tight confidence intervals;
- Shared biological function and metabolic pathways;
- Similar spatial distribution;
- Aligned temporal behavior in response to interventions.

Skin carotenoids and MPOD satisfy none of these fundamental criteria.

B. Dietary confounding undermines correlations

Diet affects the two tissues differently:

- β-carotene and lycopene strongly elevate skin carotenoid scores;
- But neither increases macular pigment.

As a result, dietary patterns can artificially inflate, obscure, or even reverse correlations, demonstrating that any observed statistical association lacks biological validity.

Correlation-based associations alone are insufficient to establish biomarker interchangeability, particularly when the biomarkers arise from distinct biological compartments and are influenced by different physiological pathways. Statistical correlations are further degraded or distorted by measurement error, heterogeneous sampling, and methodological variability, which can substantially attenuate or inflate Pearson correlation coefficients. These limitations are well recognized in the biomarker literature and underscore why correlation alone cannot justify surrogate substitution [14,15].

### 6.5. Direct Comparative Studies Show No Predictive Value

Studies that have measured both biomarkers in the same individuals consistently demonstrate that MPOD does not correlate with skin carotenoid scores at any retinal eccentricity, and total macular pigment volume shows no meaningful association with skin readings. Longitudinal data further show that the two measurements follow completely different trajectories: skin carotenoids rise within weeks, whereas MPOD increases only after months of sustained lutein and zeaxanthin intake [6,7]. Similar lack of predictive association has been reported in pediatric and school-aged cohorts, where skin carotenoid levels show little or no relationship with macular pigment measures despite shared dietary influences [16,17]. Taken together, these findings reinforce the biochemical and anatomical evidence that skin carotenoid scores cannot predict MPOD.

### 6.6. Summary of Statistical Findings

- Reported correlations between skin carotenoids and MPOD range widely (r ≈ 0.02–0.66) and are inconsistent across studies.
- Large, diverse population studies consistently show near-zero associations, indicating biological non-equivalence.
- Moderate correlations reported in small studies are likely inflated by limited demographic diversity, dietary homogeneity, and methodological constraints.
- Skin measurements reflect total carotenoids dominated by β-carotene and lycopene, whereas MPOD reflects only lutein, zeaxanthin, and meso-zeaxanthin.
- Divergent temporal responses further undermine predictive alignment between the two biomarkers.

Collectively, the statistical evidence confirms that skin carotenoid measurements cannot predict, substitute for, or serve as surrogates for macular pigment optical density.

## 7. Physiological and Environmental Factors Underscoring the Non-Equivalence of Skin Carotenoids and Macular Pigment

Beyond biochemical, anatomical, and optical distinctions, the skin and retina operate in entirely different physiological and environmental contexts. These differences strongly influence carotenoid behavior in each tissue and provide additional evidence that skin carotenoid measurements cannot be used to infer MPOD.

### 7.1. Distinct Functional Roles of Carotenoids in Skin vs. Retina

In the skin, carotenoids function primarily as broad-spectrum antioxidants that neutralize reactive oxygen species generated by ultraviolet radiation, environmental pollutants, temperature extremes, and systemic metabolic stressors [3]. Because the epidermis and dermis are continuously exposed to external influences, skin carotenoid levels fluctuate readily and often independently of dietary intake.

In the retina, the roles of lutein, zeaxanthin, and meso-zeaxanthin are far more specialized [7]. These macular carotenoids absorb high-energy blue light, quench photochemically generated radicals, protect photoreceptors and the RPE, and support visual performance and contrast sensitivity. Their deposition reflects long-term retinal metabolism and neural oxidative demand rather than environmental exposure. These tightly regulated, retina-specific functions contrast sharply with the general antioxidant protection carotenoids provide in the skin.

### 7.2. Skin Carotenoids Are Strongly Influenced by Environmental Exposure

Skin carotenoid measurements are highly sensitive to environmental and physiological fluctuations. Ultraviolet exposure increases oxidative demand and readily depletes epidermal carotenoids. Surface-level interactions, such as sunscreen application or topical antioxidants, can alter reflectance-based readings. Temperature-induced changes in dermal perfusion, hydration status, sweating, psychological stress, and even pressure applied during measurement can generate short-term variability. These factors can produce measurable changes in skin carotenoid scores within hours or days, even without any dietary change.

In contrast, the retina is insulated from the environmental variables that drive rapid fluctuations in skin carotenoids. The cornea and crystalline lens filter ultraviolet light; no topical substances interact with retinal tissue; ocular temperature remains stable; and the choroidal circulation is tightly regulated. The spatial distribution of macular pigment is determined by retinal anatomy and selective carotenoid transport mechanisms, not by external environmental conditions.

Consequently, MPOD is a stable, slowly evolving biomarker that reflects long-term neural antioxidant status, in direct contrast to the environmentally reactive behavior of skin carotenoids.

### 7.3. Differences in Carotenoid Turnover and Renewal

Epidermal turnover occurs approximately every 4 to 6 weeks, and carotenoid levels in the superficial layers decline rapidly under oxidative stress. New carotenoids are continually delivered through dermal capillaries, making the skin a dynamic reservoir with fast turnover and high sensitivity to environmental factors.

In contrast, macular pigment is located within stable retinal layers. Carotenoid turnover in the retina is slow, tightly regulated, and dependent on selective uptake mechanisms involving specialized transport proteins and cellular pathways. As a result, changes in macular pigment occur over months rather than weeks.

#### Implication

These fundamentally different turnover kinetics prevent any meaningful temporal alignment between skin carotenoid levels and retinal macular pigment.

### 7.4. Impact of Diet: Skin Captures Total Carotenoids, Retina Requires Specific Carotenoids

Skin carotenoid levels rise in response to broad dietary carotenoid intake, including β-carotene, lycopene, α-carotene, and mixed phytonutrient sources found in fruits and vegetables. These compounds dominate systemic circulation and therefore dominate skin-based measurements.

The retina, however, responds exclusively to lutein, zeaxanthin, and meso-zeaxanthin, the only carotenoids that contribute to macular pigment [7]. As a result, foods that substantially elevate skin carotenoid levels may have little or no effect on MPOD.

#### Example

Tomato juice markedly increases skin lycopene but does not increase macular pigment, underscoring the independent dietary drivers of the two biomarkers.

### 7.5. Sun Exposure vs. Light Exposure: Opposite Physiological Effects

#### 7.5.1. Skin

Ultraviolet exposure increases oxidative stress in the epidermis and rapidly depletes skin carotenoids. This environmental sensitivity is one of the dominant sources of variability in skin carotenoid measurements.

#### 7.5.2. Retina

The retina receives no ultraviolet radiation because the cornea and crystalline lens fully filter UV wavelengths. Retinal carotenoid dynamics are instead influenced by visible-light–induced photochemical stress, neural metabolism, and long-term oxidative demands within photoreceptors and the RPE.

#### 7.5.3. Implication

The environmental factor that most strongly affects skin carotenoids (UV exposure) has no role in determining MPOD. This fundamental difference further demonstrates that skin carotenoid behavior and macular pigment behavior are biologically independent.

### 7.6. Systemic vs. Neural Physiology

Skin physiology reflects whole-body influences, including dietary patterns, systemic metabolism, oxidative stress, hydration status, lifestyle behaviors, and environmental exposures. As a result, skin carotenoid levels fluctuate in response to broad changes in systemic antioxidant load and external conditions.

Retinal physiology, by contrast, is shaped by the specialized needs of neural tissue. Macular carotenoids are regulated by local photoreceptor oxidative stress, neural metabolism, and long-term selective transport involving specific binding and uptake proteins. Even when systemic carotenoid availability is high, the retina selectively accumulates only lutein, zeaxanthin, and meso-zeaxanthin according to neural antioxidant requirements.

Thus, identical dietary intake can produce completely different carotenoid behaviors in skin and retina, reinforcing that the two biomarkers are biologically independent.

### 7.7. Summary of Physiological and Environmental Evidence

- Skin carotenoid levels are strongly influenced by environmental and systemic factors such as UV exposure, temperature, hydration, stress, and vascular perfusion.
- Skin carotenoids exhibit rapid turnover and reflect broad systemic antioxidant status rather than tissue-specific function.
- Macular pigment is insulated from environmental variability and is governed by neural metabolic demand and selective retinal transport.
- Retinal carotenoid turnover is slow and tightly regulated, in contrast to rapid epidermal renewal.
- These physiological and environmental differences further demonstrate that skin carotenoid measurements cannot substitute for MPOD.

## 8. Why Skin Carotenoid Measurements Cannot Serve as a Proxy for MPOD

Evidence across biochemical, anatomical, optical, physiological, temporal, and statistical domains shows that skin carotenoid measurements and MPOD represent fundamentally different biological systems. Although both tissues store carotenoids and respond to dietary intake, their mechanisms of transport, selective uptake, turnover, and functional roles differ so profoundly that the two biomarkers cannot be equated. The following sections integrate these findings to explain why skin carotenoid scores cannot serve as surrogate markers for MPOD [9,10,11,12].

### 8.1. Different Carotenoid Molecules Are Being Measured

Skin and retinal tissues accumulate different carotenoid species because each tissue expresses distinct transporters and serves different physiological functions. Skin carotenoid measurements primarily capture β-carotene, lycopene, α-carotene, and β-cryptoxanthin, with only small contributions from lutein and zeaxanthin. These carotenoids dominate systemic circulation and reflect general fruit and vegetable intake [18,19,20].

The macula, however, contains only lutein, zeaxanthin, and meso-zeaxanthin. These xanthophylls are the sole contributors to blue-light filtration, foveal oxidative protection, and visual performance. Their selective enrichment in the retina is driven by specialized binding and transport proteins that are not expressed in the skin.

Because the two tissues accumulate fundamentally different carotenoid species, a skin measurement cannot provide meaningful information about the xanthophylls that determine MPOD. This biochemical mismatch alone is sufficient to rule out the use of skin carotenoid scores as a proxy for macular pigment.

### 8.2. Skin and Retina Have Opposite Spatial Organization

The spatial distribution of carotenoids differs fundamentally between skin and retina. In the skin, carotenoids are spread diffusely across a broad sampling area and are intermixed with melanin, hemoglobin, and collagen. This distribution is uniform and does not form any anatomically localized pigment concentration.

In contrast, macular pigment is tightly confined to the central 1–2 mm of the retina and follows distinct spatial profiles, including central peak, ring-like, or asymmetric patterns. These phenotypes arise from the unique structure of the fovea and carry important functional and clinical significance. Because MPOD depends on this precise, localized organization, a single global skin carotenoid value cannot provide meaningful information about macular pigment distribution.

### 8.3. Optical Properties Are Fundamentally Incompatible

The optical environments of the skin and retina differ in ways that make their carotenoid signals impossible to compare. In the skin, optical measurements are shaped by epidermal melanin, dermal hemoglobin, collagen-associated scattering, and shallow penetration depth. These interactions generate a surface-weighted signal composed of multiple chromophores rather than a discrete carotenoid layer.

Retinal imaging operates in a completely different optical setting. Light must traverse several neural layers, interact with macular pigment, pass through the melanin-rich retinal pigment epithelium, and encounter lipofuscin autofluorescence and choroidal vasculature. These structures create depth-dependent, layered optical pathways that require wavelength-specific corrections and anatomical modeling. Because the optical physics governing retinal imaging has no analogue in the skin, no skin-based method can approximate or reconstruct the layered pigment absorption that defines MPOD.

### 8.4. Environmental and Physiological Influences Are Entirely Different

Skin carotenoid concentrations fluctuate in response to numerous environmental and physiological variables, including ultraviolet exposure, temperature, hydration, vascular perfusion, autonomic responses, and surface-level interactions such as sunscreen or topical antioxidants. These factors can change skin carotenoid scores independently of dietary intake.

In contrast, macular pigment levels change only through sustained intake of lutein and zeaxanthin and are shaped by retinal oxidative demand, neural metabolism, and long-term selective transport. Environmental factors that strongly influence skin carotenoids have no effect on the retina. This divergence underscores a fundamental physiological disconnect between the two tissues.

### 8.5. Temporal Dynamics Do Not Align

Temporal responses to carotenoid intake further demonstrate the separation between skin and retina. Skin carotenoids typically rise within 2 to 4 weeks and often plateau by 6 to 8 weeks. MPOD, however, shows little or no measurable change during the first 8 weeks and then increases gradually over the next 4 to 6 months, with significant improvements often requiring 6 to 12 months of sustained intake.

These opposing timelines make it biologically impossible for skin carotenoid scores to predict macular pigment levels at any stage of supplementation or dietary change. The biomarkers reflect different physiological mechanisms and operate on fundamentally different timescales.

### 8.6. Statistical Evidence Confirms Biological Separation

Across the published literature, statistical correlations between skin carotenoid levels and MPOD are weak, inconsistent, and highly dependent on study design. Large cohorts consistently show near-zero correlations, while moderate correlations in small studies are frequently inflated by methodological constraints, limited demographic diversity, or restricted dietary variation. Correlations also collapse when dietary patterns include carotenoids such as β-carotene or lycopene, which strongly elevate skin measurements but have no effect on MPOD.

In biomarker science, such weak and unstable associations cannot justify surrogate use. The statistical evidence, therefore, reinforces the underlying biological separation between skin and retinal carotenoid systems.

### 8.7. Retinal-Specific Mechanisms Cannot Be Accessed from Skin

Macular pigment deposition depends on retina-specific uptake pathways involving transport proteins such as SCARB1, STARD3, and GSTP1, deposition within defined retinal layers, and imaging methods that correct for melanin and lipofuscin. None of these biological or optical mechanisms has any counterpart in the skin. Because the physiological machinery that determines MPOD exists solely within the retina, it cannot be inferred, either directly or indirectly, from any skin-based measurement.

### 8.8. Conclusion of Section 8

- Skin carotenoid measurements and MPOD represent fundamentally different biological systems across all examined domains.
- Skin measurements reflect broad systemic carotenoid exposure dominated by β-carotene and lycopene.
- MPOD reflects selective retinal accumulation of lutein, zeaxanthin, and meso-zeaxanthin within neural tissue.
- Anatomical localization, optical environments, physiological regulation, and temporal dynamics differ profoundly between skin and retina.
- Consequently, skin carotenoid scores cannot estimate, predict, or substitute for macular pigment optical density.

## 9. Implications for Nutrition Research, Clinical Practice, and Public Health

The evidence presented in this review demonstrates that skin carotenoid measurements and MPOD reflect fundamentally different biological systems. They respond to different carotenoid classes, reside in tissues with distinct anatomical and optical environments, and are shaped by entirely different physiological and environmental influences. These differences carry important implications for how each biomarker should be interpreted in nutrition research, ophthalmology, wellness technologies, and public health communication.

### 9.1. Implications for Nutrition and Dietary Intervention Research

Skin carotenoid scores are valuable short-term indicators of fruit and vegetable intake. Because they primarily reflect β-carotene, lycopene, α-carotene, and cryptoxanthin [3], they are effective for assessing adherence to dietary interventions, monitoring changes in general antioxidant intake, evaluating population-level nutrient status, and supporting community nutrition programs. In these settings, skin measurements function as convenient, non-invasive biomarkers of systemic dietary carotenoid exposure.

However, their relevance does not extend to retinal carotenoid biology. Skin carotenoid scores do not assess lutein or zeaxanthin levels in the retina and cannot substitute for MPOD in studies examining visual function or ocular health. They should not be used to infer AMD risk, retinal oxidative protection, or visual performance, and they cannot validate the effectiveness of supplementation intended to increase macular pigment.

### 9.2. Implications for Vision Science and Ophthalmology

MPOD is a retina-specific biomarker directly linked to visual and neural health. It contributes to blue-light filtration, photoreceptor protection, modulation of AMD risk, and improvements in contrast sensitivity and visual performance [21]. Because the retina selectively accumulates lutein, zeaxanthin, and meso-zeaxanthin, accurate assessment of macular health requires direct retinal measurement [1,6,8].

Skin carotenoid scores provide no clinically meaningful information about retinal carotenoid status. They cannot detect macular pigment deficits, reveal spatial distribution patterns such as peak, ring, or asymmetric profiles, or indicate risk for retinal disease. They also cannot monitor clinical response to lutein or zeaxanthin supplementation.

Therefore, direct retinal imaging remains essential for both research and clinical ophthalmology.

### 9.3. Implications for Consumer Wellness Devices and Commercial Claims

As skin carotenoid measurement devices become more common in consumer wellness markets, some platforms have suggested that increases in skin carotenoid scores reflect improved “eye health” or higher “macular antioxidant” levels. The scientific evidence reviewed in this manuscript does not support such interpretations.

Skin carotenoid changes often reflect increases in β-carotene or lycopene intake, which do not affect MPOD. These measurements are also sensitive to ultraviolet exposure, temperature, hydration status, stress, and epidermal turnover, none of which meaningfully influence retinal physiology. Therefore, increases in skin carotenoid scores do not imply increases in macular pigment, and decreases do not indicate retinal deficiency or elevated AMD risk.

A clear distinction between systemic antioxidant biomarkers (skin) and retinal protection biomarkers (macula) is essential for ethical and evidence-based consumer communication. Claims should remain tissue-specific to avoid misleading users.

### 9.4. Implications for Public Health and Education

Public misunderstanding can arise when biomarkers are interpreted without a biological context. Public health communication should therefore emphasize the distinction between skin carotenoid levels and retinal macular pigment. Clear messaging reduces confusion about “eye health,” supports evidence-based nutritional guidance, and helps communities understand that dietary carotenoids and macular pigment serve complementary but non-interchangeable roles.

Skin carotenoid scores remain useful for assessing general nutritional status, but they cannot replace macular pigment assessment in populations at risk for retinal disease.

### 9.5. Implications for Supplementation Programs

Skin carotenoid measurements can be helpful in monitoring adherence to broad dietary interventions or mixed carotenoid supplementation programs. They are well-suited for evaluating compliance with antioxidant-rich diets and tracking population-level changes in overall carotenoid exposure.

However, they cannot evaluate lutein or zeaxanthin uptake, quantify retinal deposition of macular carotenoids, or assess supplement effectiveness for visual or cognitive outcomes. Determining whether retinal carotenoid needs are being met requires direct MPOD assessment through retinal imaging.

### 9.6. Summary

- Skin carotenoid measurements are appropriate biomarkers of general dietary carotenoid intake and systemic antioxidant exposure.
- MPOD is a retina-specific biomarker that requires direct retinal imaging for accurate assessment.
- Substituting skin carotenoid scores for MPOD risks misleading conclusions in nutrition research, clinical practice, and consumer wellness contexts.
- A clear distinction between systemic and retinal carotenoid biomarkers is essential for evidence-based communication and intervention design.

## 10. Conclusions

Carotenoids play important biological roles throughout the human body, yet their functions, anatomical locations, optical environments, and temporal behavior differ fundamentally between the skin and the retina. Evidence across biochemical, anatomical, optical, physiological, and statistical domains demonstrates that skin carotenoid measurements cannot serve as proxies for MPOD [1,3,4,9].

Skin carotenoids primarily represent β-carotene, lycopene, α-carotene, and cryptoxanthin. They accumulate diffusely within superficial skin layers and respond rapidly to environmental factors and short-term dietary intake. In contrast, MPOD reflects the selective deposition of lutein, zeaxanthin, and meso-zeaxanthin within the neurosensory retina, governed by specialized transport and binding mechanisms that operate over long time scales.

These distinctions are reinforced by several fundamental differences:

- Optical environments:
  Skin signals arise from interactions with epidermal melanin and dermal hemoglobin, whereas retinal imaging must account for macular pigment absorption, RPE melanin, and lipofuscin autofluorescence.
- Temporal kinetics:
  Skin carotenoids typically change over weeks, whereas MPOD increases require months or longer.
- Environmental influences:
  Skin carotenoids fluctuate with ultraviolet exposure, temperature, hydration, and perfusion, none of which meaningfully affect retinal carotenoid status.
- Statistical independence:
  Large, methodologically robust studies consistently report weak or negligible correlations between skin carotenoid scores and MPOD.

Taken together, the cumulative evidence shows that although skin carotenoid measurements provide valuable insight into general dietary carotenoid intake and systemic antioxidant exposure, they cannot inform retinal carotenoid status, macular pigment distribution, visual function, or retinal health.

Therefore:

Direct retinal imaging is required for accurate assessment of macular pigment optical density.

Skin carotenoid scores cannot substitute for, estimate, or predict MPOD under any scientific, nutritional, wellness, or clinical circumstance.

A clear understanding of tissue-specific carotenoid biology is essential for the correct interpretation of these biomarkers across nutrition research, ophthalmology, consumer health technology, and public health communication. Maintaining this distinction will help prevent misconceptions and support accurate conclusions regarding retinal health.

## Figures and Tables

**Table 1:** Qualitative comparison of carotenoid presence and selective bioavailability in human skin versus the macula. This table provides a qualitative, presence-based comparison of major dietary carotenoids in skin and macular tissue. It is not intended to report absolute concentrations, which vary widely across studies depending on analytical method, tissue layer, population, and sampling conditions. Skin carotenoids reflect non-selective systemic accumulation dominated by β-carotene and lycopene, whereas the macula selectively accumulates lutein and zeaxanthin and contains meso-zeaxanthin formed locally in the retina via enzymatic conversion of lutein. These tissue-specific differences in carotenoid selectivity underlie the non-equivalence of skin carotenoid measurements and MPOD.

Carotenoid	Skin (Systemic Accumulation)	Macula (Selective Neural Uptake)
β-carotene	Present; commonly dominant	Not accumulated
Lycopene	Present; commonly dominant	Not accumulated
α-carotene	Present	Not accumulated
β-cryptoxanthin	Present	Not accumulated
Lutein	Present; increases with intake	Accumulated selectively when available
Zeaxanthin	Present at low levels; increases with intake	Accumulated selectively when available
Meso-zeaxanthin	Not accumulated	Formed in the retina via conversion of lutein

## Data Availability

No new data were created or analyzed in this study.
